# Supplementary material for: Effects of the Calix[4]arene Derivative Compound OTX008 on High Glucose-Stimulated ARPE-19 Cells: Focus on Galectin-1/TGF-β/EMT Pathway
Source: Molecules. 2022 Jul 26;27(15):4785. doi: 10.3390/molecules27154785 (PMC9332238; doi:10.3390/molecules27154785)
Supplement: Supplementary file 1 [file molecules-27-04785-s001.zip › molecules-1813471-supplementary.pdf]

molecules

Effects of the Calix [4]arene Derivative Compound OTX008 on High Glucose-Stimulated ARPE-19 Cells: Focus on Galectin-1/TGF-β/EMT Pathway

Maria Consiglia Trotta [1], Francesco Petrillo [2], Carlo Gesualdo [3], Settimio Rossi [3], Alberto Della Corte [3], Judit Váradi [4], Ferenc Fenyvesi [4], Michele D'Amico [1] and Anca Hermenean [5,*]

[1] Department of Experimental Medicine, University of Campania "Luigi Vanvitelli", 80138 Naples, Italy; mariaconsiglia.trotta2@unicampania.it (M.C.T.); michele.damico@unicampania.it (M.D.)
[2] PhD Course in Translational Medicine, Department of Experimental Medicine, University of Campania "Luigi Vanvitelli", 80138 Naples, Italy; francescopetrillo09@gmail.com
[3] Multidisciplinary Department of Medical, Surgical and Dental Sciences, Eye Clinic, University of Campania "Luigi Vanvitelli", 80138 Naples, Italy; carlo.gesualdo@unicampania.it (C.G.); settimio.rossi@unicampania.it (S.R.); albertodellacorte@live.it (A.D.C.)
[4] Department of Pharmaceutical Technology, Faculty of Pharmacy, University of Debrecen, Nagyerdei St. 98, H-4032 Debrecen, Hungary; varadi.judit@pharm.unideb.hu (J.V.); fenyvesi.ferenc@pharm.unideb.hu (F.F.)
[5] Faculty of Medicine, Vasile Goldis Western University of Arad, 310414 Arad, Romania
* Correspondence: hermenean.anca@uvvg.ro

SUPPLEMENTARY MATERIALS

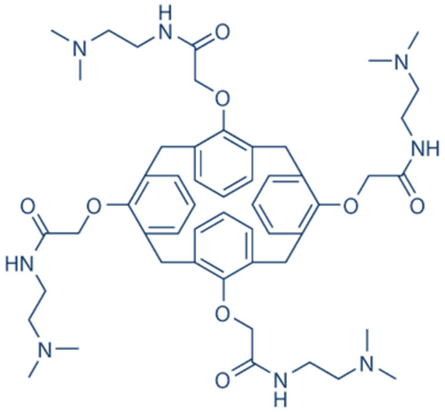

Figure S1. OTX008 chemical structure (as reported by S6949, Selleckchem, Houston, TX 77014 USA).

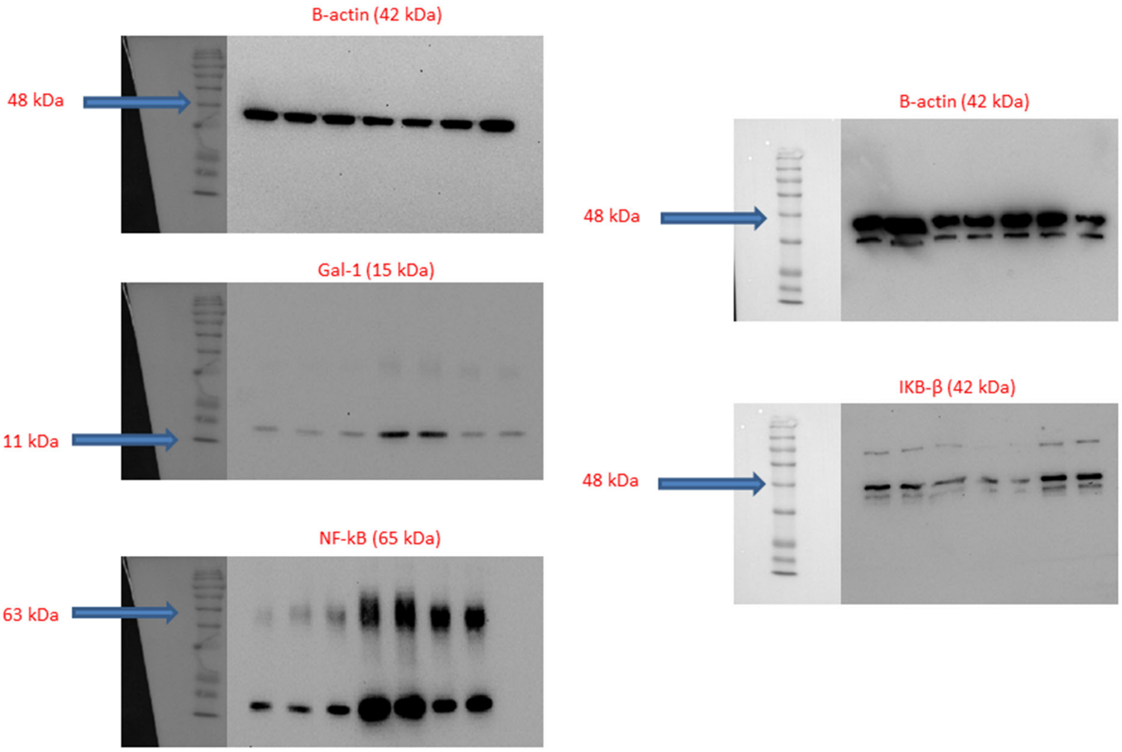

Figure S2. Uncropped images of representative β-actin (to mouse), Gal-1 (to mouse), NF-kB (to rabbit), IkB-β (to rabbit) and β-actin (to mouse) Western Blotting membranes.
